# The Oxygen Reduction Reaction Rate of Metallic Nanoparticles during Catalyzed Oxidation

**DOI:** 10.1038/s41598-017-07717-4

**Published:** 2017-08-01

**Authors:** Ke Sun, Jinbo Xue, Kaiping Tai, Shen J. Dillon

**Affiliations:** 1 Department of Materials Science and Engineering, University of Illinois Urbana-Champaign, Urbana, IL USA; 2 Department of Materials Science and Engineering, Taiyuan University of Technology, Taiyuan, China; 3 Functional Films and Interfaces Division, Shenyang National Laboratory for Materials Science, Institute of Metal Research Chinese Academy of Sciences, Shenyang, China

## Abstract

This work reports the oxygen reduction reaction (ORR) kinetics of metal nanoparticle catalysts between 500 and 600 °C at low oxygen partial pressures. *Ex situ* and *in situ* TEM measurements demonstrate catalyzed nanowire growth initially follows linear kinetics; characteristic of being ORR rate limited. The ORR rates of Ag, Au, Cu, Ni, Pd, Rh and Pt measured at 600 °C form a volcano plot versus relative oxidation potential. Cu nanoparticles produce the maximum ORR rate under these conditions.

## Introduction

Oxygen reduction reaction (ORR) kinetics have generated great interest due to their rate limiting role in fuel cells and metal-air batteries^1–4^. ORR kinetics have primarily been investigated computationally or through electrochemical measurements^1–3, 5–8^. Many experimental investigations focus on low temperature behavior and have focused on noble metal ORR catalysts for H_2_ oxidation. Pt catalyst is ideal for this application, and great effort has been dedicated to understanding the reaction mechanisms in effort to discover low cost alternatives^5, 7, 9–13^. Pt’s efficacy in promoting the ORR derives from an optimization between the activation energies for O_2_ binding to the catalyst and debinding of the reaction product^2, 14^. This leads to the well know ‘volcano plot’ behavior, where an optimum lies at a specific potential. Volcano plots have been calculated for a range of ORR metal catalysts, but experimental measurements have primarily been obtained from noble metals^2, 11, 14–17^. Aqueous electrochemical measurements at ambient pO_2_ oxidize most non-noble metal nanoparticles leaving a gap in our experimental understanding.

In considering alternative ORR limited systems, such as metal-air batteries, the wealth of knowledge gained from fuel cell research provides a basis for catalyst selection. However, in such systems catalysts such as Cu that function poorly in fuel cells have been shown to perform quite well^18^. Pd similarly outperforms Pt in non-aqueous cells^15^. The ORR in metal-air batteries forms metal oxide, peroxide, or carbonate, which have large formation energies^3, 19, 20^. The impact of having a solid oxide reaction product on the optimization of catalyst chemistry has received limited experimental attention.

A novel experimental approach utilized here exploits direct measurement of solid phase reaction product volume to provide accurate ORR rates. We previously demonstrated ORR catalysts utilized to locally accelerate the oxidation rate of an underlying metallic substrate can promote nanowire growth forming a variety of metal oxide nanowires^21^. In this work, the ORR kinetics of different pure metal nanoparticle catalysts are investigated using iron oxidation as a model system. Iron is ideal since it can effectively reduce a number of other transition metal oxides, and Fe_3_O_4_ has a similar formation energy, per mole of O_2_, as water.

**Figure 1:**
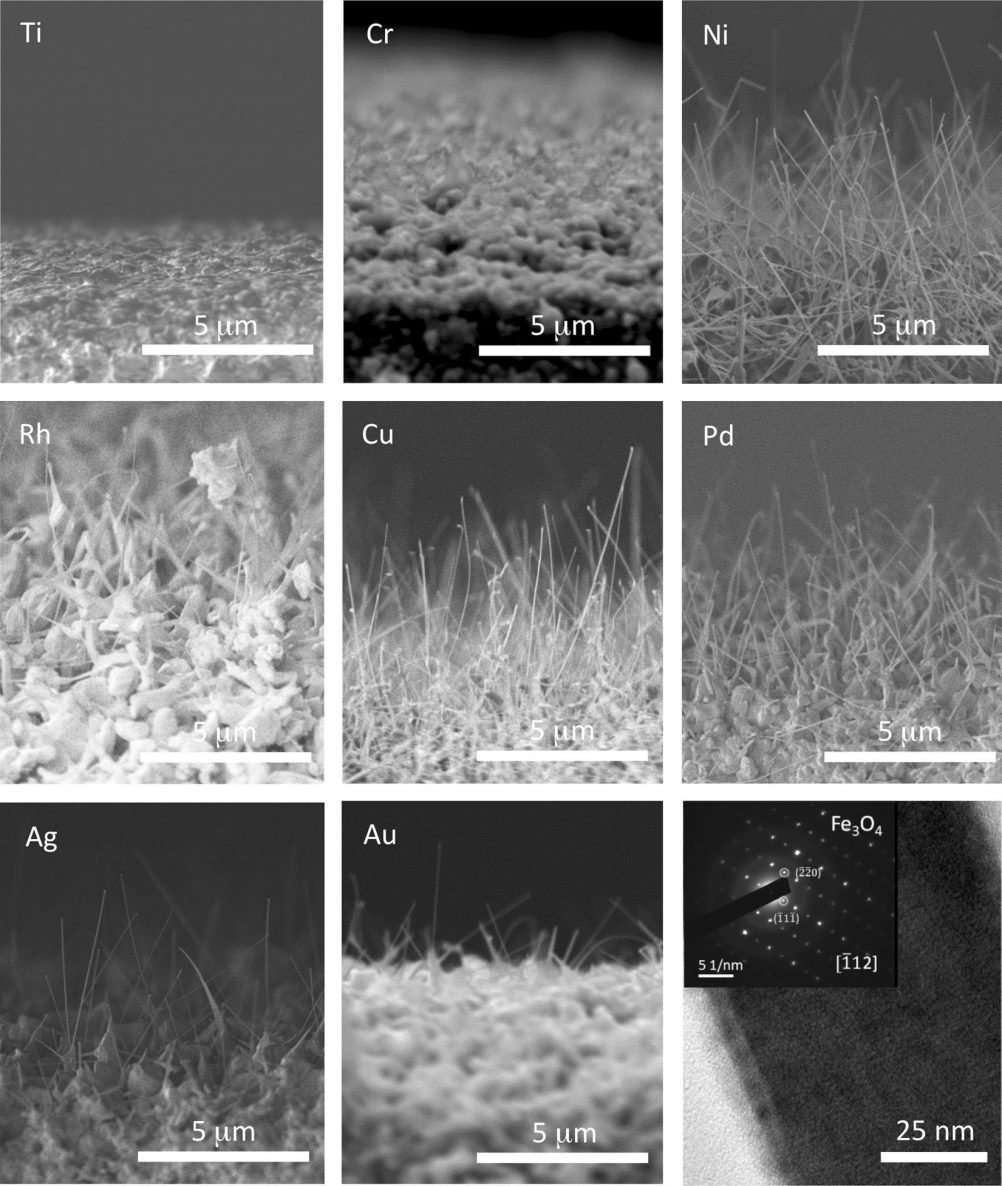
SEM images of samples where Ti, Cr, Ni, Rh, Cu, Pd, Ag, and Au were used as catalysts for nanowire growth at 600 °C and 100 ppm O_2_. Ti and Cr catalyst did not produce nanowires. The image in the lower right shows a TEM image of a nanowire along with electron diffraction inset.

Ag, Au, Cu, Ni, Pd, Rh and Pt catalysts deposited on a 200 nm MgO coated Fe substrate promoted nanowire growth at 600 °C and 100 ppm O_2_. Ti and Cr did not promote nanowire growth (Fig. 1) under the same conditions. Ti and Cr are not anticipated to function as an effective ORR catalyst for Fe_3_O_4_ nanowire growth, because they have a higher O_2_ affinity than Fe. We confirmed via TEM imaging and STEM EDS that each nanowire indeed terminates with a metal nanoparticle (Fig. S1), found that nanowires in each case are composed of Fe_3_O_4_, and observed that all long NWs analyzed grow in the [110] direction (Fig. 1).

**Figure 2:**
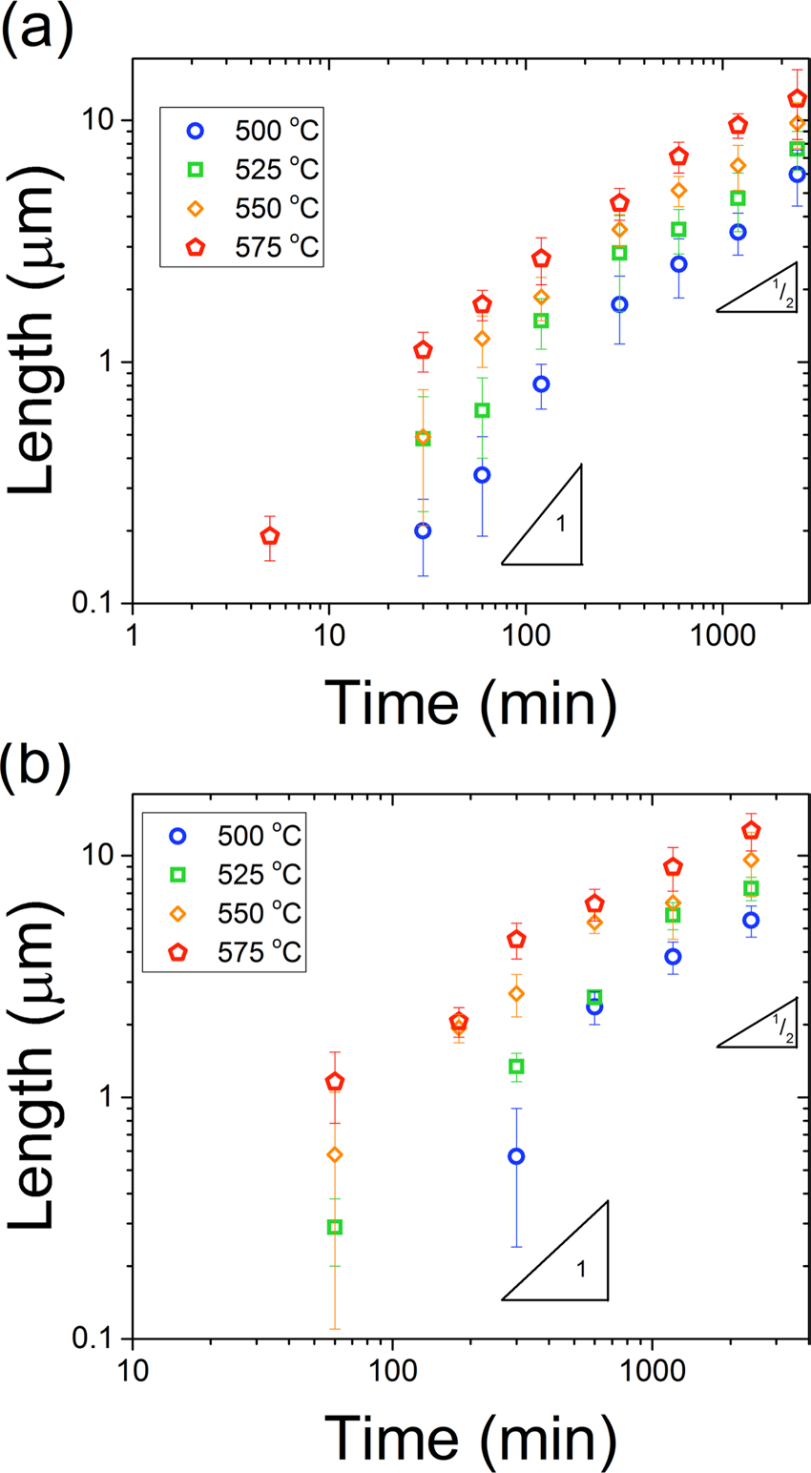
Growth kinetics of (**a**) Cu catalyzed and (**b**) Pd catalyzed nanowire growth at different temperatures.

Pd and Cu were investigated in detail, *ex situ,* in order to characterize the kinetic regimes as a function of time and temperature. Linear kinetics occur at shorter times, and growth transitions to parabolic kinetics at longer times (Fig. 2a and b). Linear kinetics indicate interface limited reactions, while parabolic results from diffusion kinetics^22^. The temperature dependencies of the linear and parabolic regimes are consistent with this interpretation; the activation energies (1.0 kJ/mol) for the parabolic regime are the same regardless of chemistry, but the activation energies for the linear regime are not (0.9 eV and 1.3 eV for Pd and Cu, respectively, see Supplementary Fig. S2).

**Figure 3:**
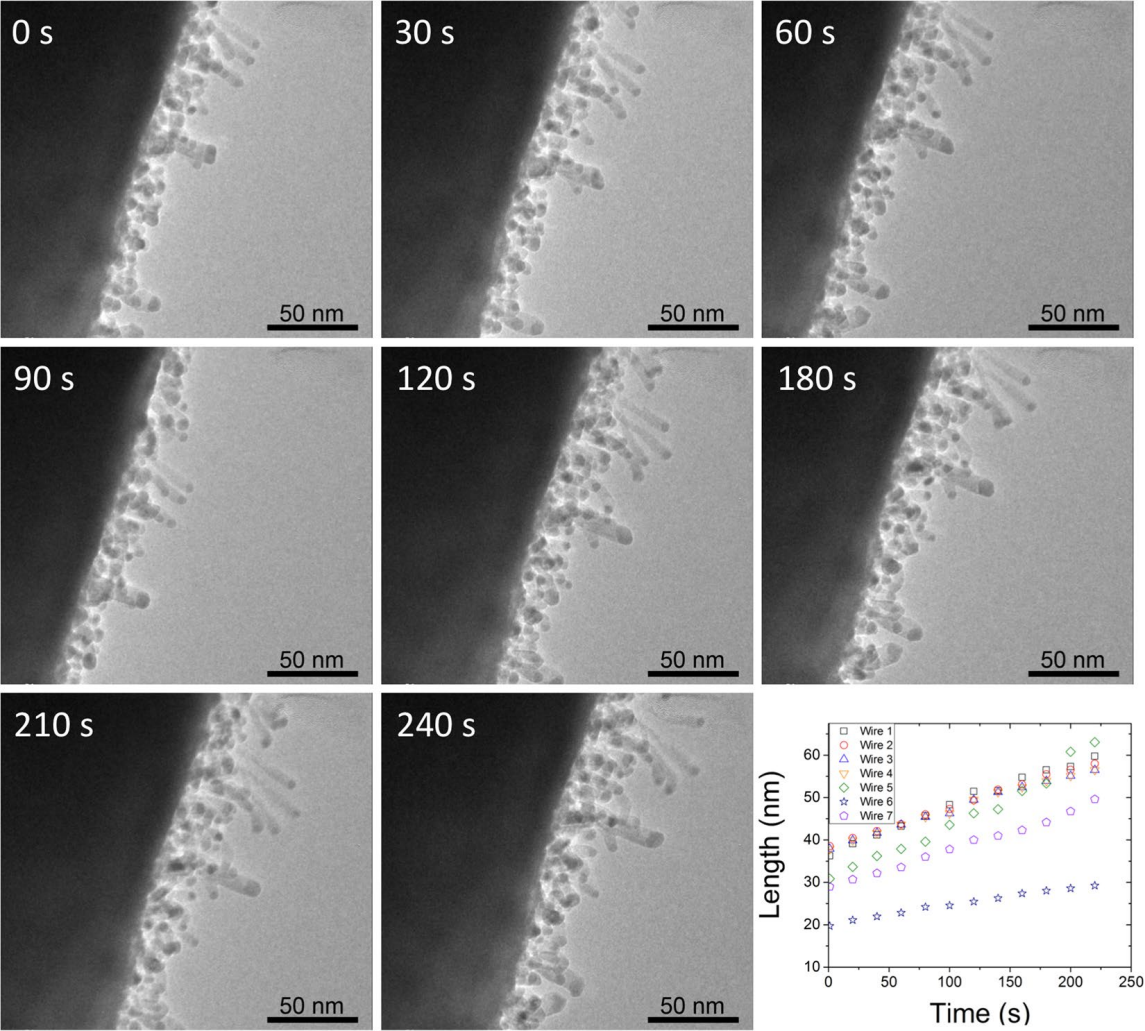
Time-lapse *in situ* TEM images of nanowire growth at ca. 500 °C. The growth kinetics of several single nanowires are plotted in the lower right.

To confirm the linear nature of the growth kinetics, for short wires, at the single nanowire level we performed *in situ* catalyzed oxidation experiments in the TEM. *In situ* TEM based growths were performed at a nominal average temperature of 500 °C using Pd catalyst. Figure 3 shows time-lapse images and growth kinetics associated with Fe_3_O_4_ nanowire growth catalyzed by Pd nanoparticles at ≈500 °C. The local kinetics and growth direction can be are sensitive to catalyst nanoparticle faceting and dihedral angles (Supplementary Fig. S3). Such effects are also present in electrochemical measurements, but are typically averaged together to provide a single rate. Regardless, the kinetics of individual nanowires are linear during initial nanowire growth (<≈2 μm), supporting our conclusion that kinetics are interface reaction rate limited (i.e. ORR) in this regime. The average growth rates measured *in situ* at 500 °C (≈5 nm min^−1^) are comparable to those measured at 500 °C *ex situ* (≈4 nm min^−1^), but this may be fortuitous due the limited control of local pO_2_ in the *in situ* experiment.

**Figure 4:**
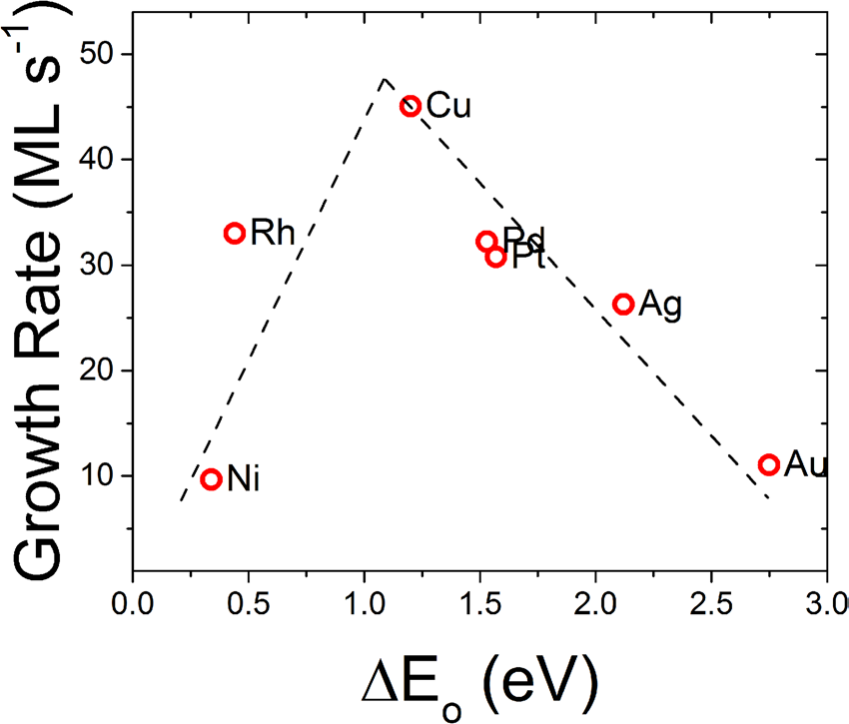
The growth rate of nanowires under different metal nanoparticle catalysts plotted as monolayers of O^2−^ in Fe_3_O_4_ per second versus the standard oxygen binding energy on the catalyst. Here a monolayer is assumed to be 15.47 atoms nm^−2^ with a O^2−^ planar spacing of 3.93 nm^−1^.

The ORR kinetics were compared across various nanoparticle chemistries by measuring the *ex situ* growth rate of the nanowires in the linear oxidation regime at 600 °C. The catalyst particle shapes and sizes are dictated by the dewetting thermodynamics and kinetics and on average differ for each catalyst chemistry. The radius ratios of the catalysts and nanowires differed between chemistries, so a normalization factor based on this ratio was applied to provide a ORR per unit catalyst surface area. Figure 4 plots the growth rate versus the standard oxygen binding energy of the catalysts. The data exhibits the typical ‘volcano plot’ shape with a maximum ORR rate observed between Cu and Rh. The ORR kinetics on Pt are considerably slower and suggest that Pt is not the optimal material in this case. The original computational work by Norskov *et al*. does not predict a significant temperature dependence to the shape of the volcano plot, since differences in temperature dependence would only appear in the pre-factor, based on their analysis^2^. However, the experimental activation energies do differ with catalyst chemistry, suggesting this could partially account for the enhanced relative Cu ORR kinetics at 500 °C. At 100 ppm, the oxygen binding energy for the reaction ½ O_2_ + 2e^−^ = >O^2−^ is shifted 0.34 eV relative to its standard value. The formation energy of Fe_3_O_4_ is 0.2 eV more exothermic than H_2_O. Both of these factors enhance the driving force for oxygen debinding from the catalyst. The standard binding energies of oxygen on Pt and Cu differ by 0.37 eV. The large entropic driving force for oxygen debinding at low partial pressure as well as the larger formation energy of Fe_3_O_4_, relative to H_2_O, will both enhance oxygen debinding kinetics shifting the peak in the volcano plot towards metals with higher oxygen affinity. These factors explain the experimentally observed maximum ORR kinetics of Cu and suggest the optimal pure metal catalyst under our experimental conditions should lie on the volcano plot between Cu and Rh (e.g. predicted to be Ir; ΔE_o_ = 1.0 eV). This work demonstrates why Pt will not be the ideal ORR catalyst in all applications, especially in reactions forming solid phases, those performed at low oxygen partial pressures, or those with more exothermic formation enthalpies. This is consistent with experimental results showing Cu and Pd ORR catalysts can outperform platinum in metal-air batteries^15^.

## Methods

Fe pellets (99.95%, Kurt J. Lesker) were polished and cleaned in acetone. Prior work on catalyzed oxidation for nanowire growth indicated that the presence of an oxide layer separating the catalyst was critical in enabling the nanowire growth, since it prevented co-oxidation of the metal and the catalyst particle. A 200 nm MgO layer was e-beam evaporated onto the polished Fe surface. MgO was selected due to its relatively high cation diffusivity and the high miscibility of Fe^2+/3+^ in MgO. Ag, Au, Cr, Cu, Ni, Pd, Pt Rh, and Ti were all deposited on the substrate to an average thickness of ≈3 nm, as measured by a quartz crystal microbalance, via physical vapor deposition. For Rh catalyst, 1 mg of Rhodium(III) acetylacetonate (Sigma Aldrich) was first dissolved in 10 ml of acetone, and then solution was deposited onto the MgO coated Fe substrate. The amount of solution utilized was intended to produce a similar amount of reduced Rh catalyst as the vapor deposited materials. Samples were annealed in a tube furnace under flowing 100 ppm O_2_ at temperatures between 500 and 600 °C for times between 0 and 2500 mins.

*In situ* TEM (Hitachi 9500) was also performed using a calibrated W heating wire and the injection of O_2_ and N_2_ at a 1 to 10 ratio with an overall flow rate of 0.33 mL min^−1^ and a gas pressure of ≈2 × 10^−2^ Pa. Fe metal was electrodeposited onto the W wires from a solution of Fe(NH_4_)_2_(SO_4_)_2_ that were pre-oxidized at 250 °C for 30 min under ambient conditions, and then coated with PVD Pd nanoparticles.

## Electronic supplementary material

Supplementary Video

Supplementary Information
