# Supplementary material for: The Oxygen Reduction Reaction Rate of Metallic Nanoparticles during Catalyzed Oxidation
Source: Sci Rep. 2017 Aug 1;7:7017. doi: 10.1038/s41598-017-07717-4 (PMC5539166; doi:10.1038/s41598-017-07717-4)
Supplement: Supplementary file 2 — Supplementary Information [file 41598_2017_7717_MOESM2_ESM.pdf]

Supporting Information: The Oxygen Reduction Reaction Rate of Metallic Nanoparticles during Catalyzed Oxidation

Ke Sun[1], Jinbo Xue[2], Kaiping Tai[3], and Shen J. Dillon[1,z]

[1]*Department of Materials Science and Engineering, University of Illinois Urbana-Champaign, Urbana, IL, USA*

[2]*Department of Materials Science and Engineering, Taiyuan University of Technology, Taiyuan, China*

[3]*Functional Films and Interfaces Division, Shenyang National Laboratory for Materials Science, Chinese Academy of Sciences*

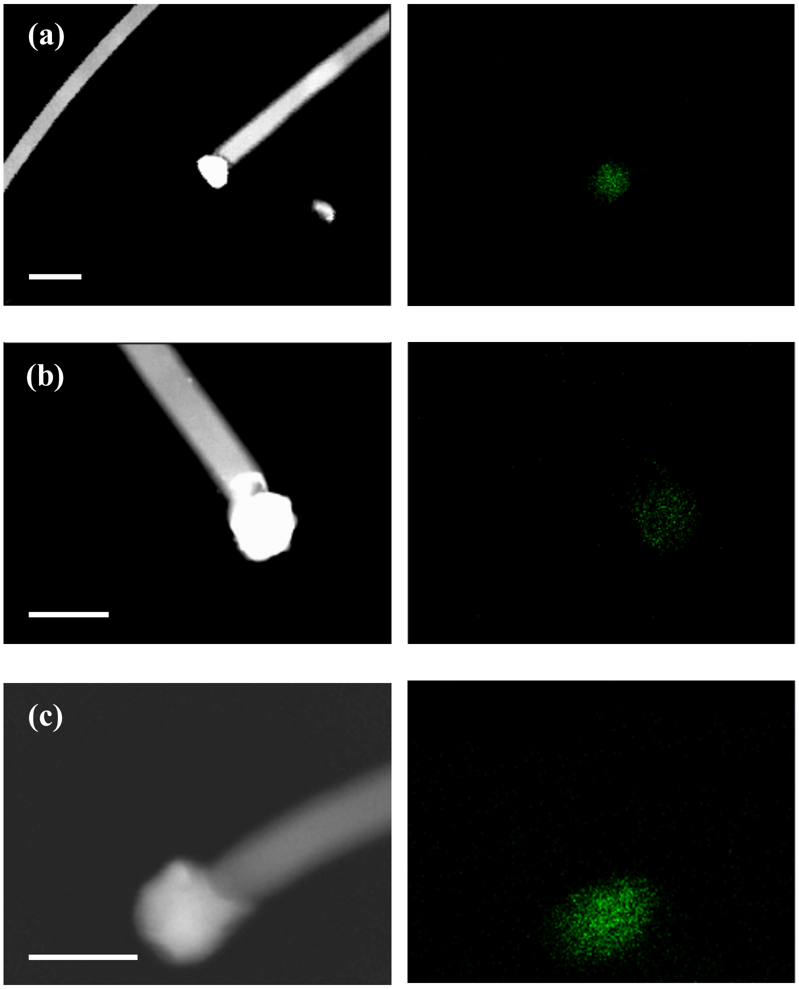

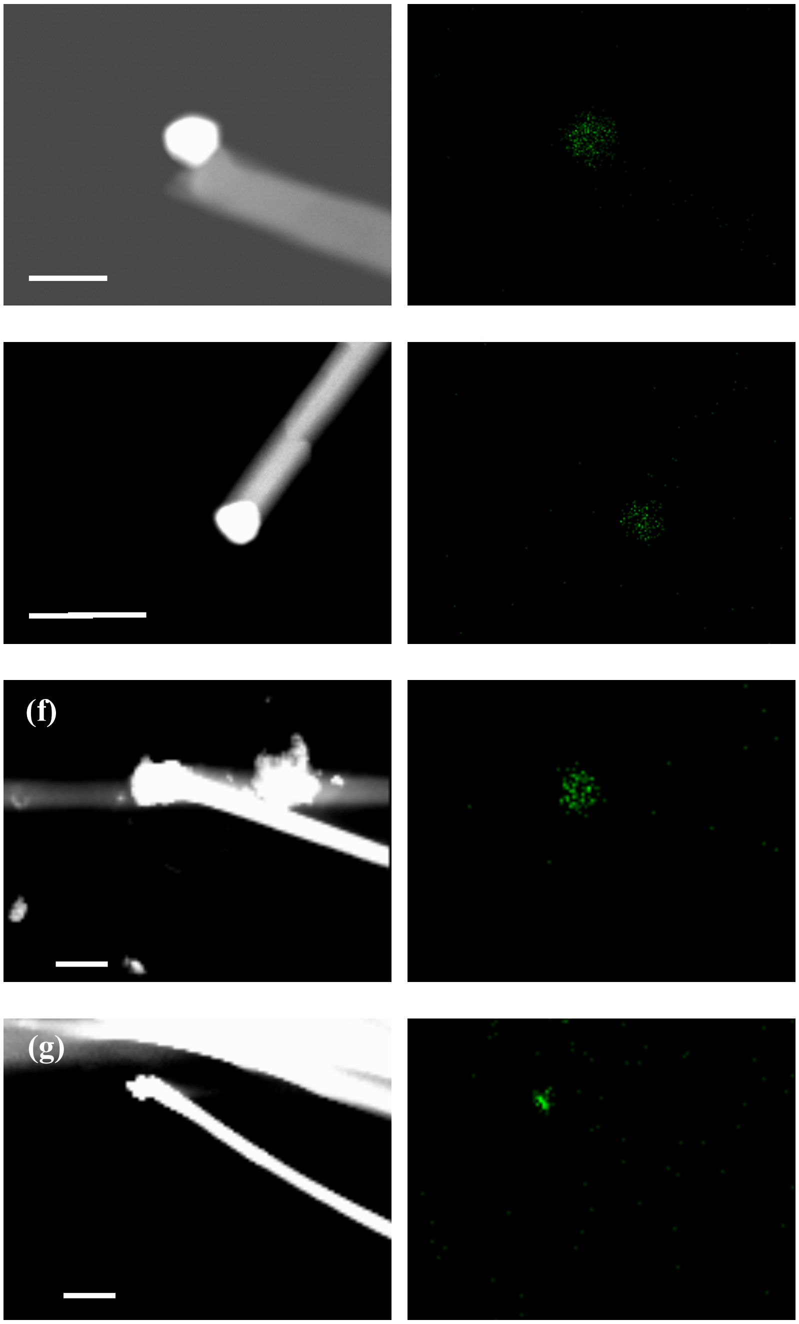

Figure S1: STEM micrographs and EDS mapping of corresponding catalysts of individual nanowires grown with different catalysts. **(a)-(g)** Ni, Ag, Cu, Au, Pd, Rh, Pt. Scale bar=100nm.

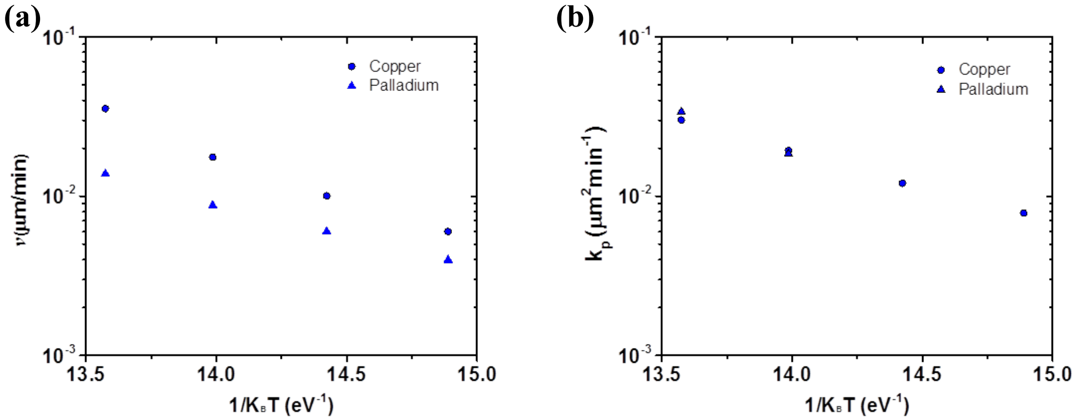

Figure S2: (a) variation of linear growth coefficients of Cu and Pd catalyzed growth process as a function of temperature. **(b)** variation of parabolic growth coefficients of Cu and Pd catalyzed growth process as a function of temperature.

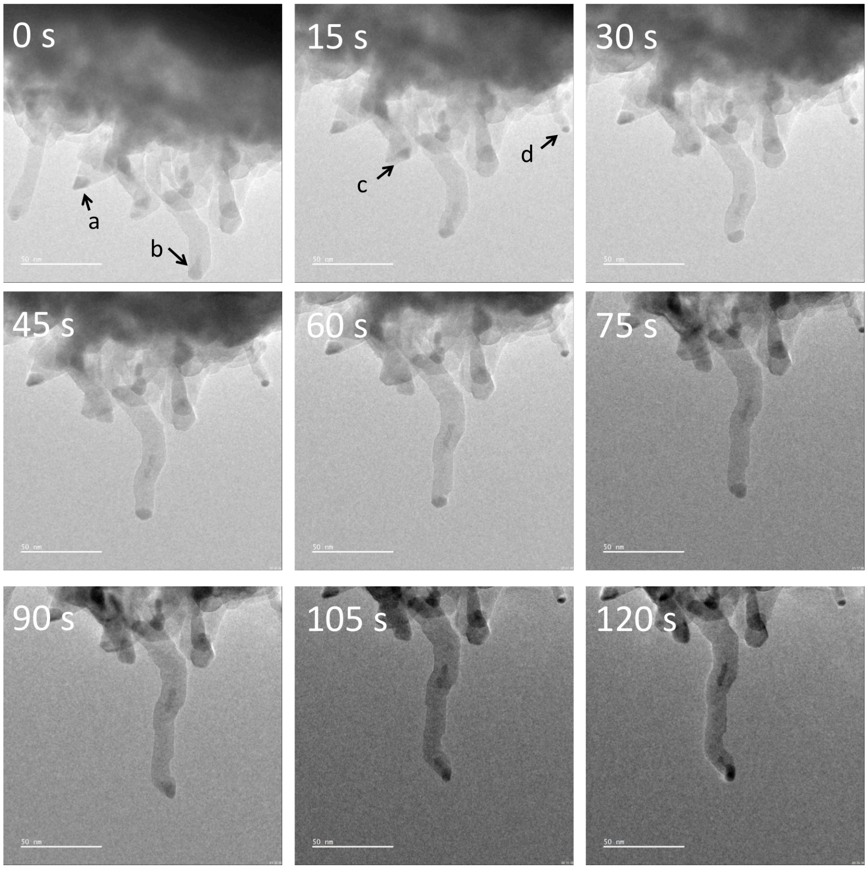

Figure S3: Time-lapse images from in-situ growth highlighting early stage nanowire development and the effect of particle geometry. Note that particle a begins with a triangular geometry whose sides are continuous with the Fe_3O_4. This particle does not

grow appreciably, as further elongation would require a change in the wetting condition. A similar effect is observed for particle b at longer times. Particle b initially grows rapidly after the Pd nanoparticle partially dewets, leaving behind Pd either on the surface or in the lattice. The round particle facilitates rapid growth. However, at around 75 s, the particle takes on a more tirangular morphology, that causes the nanowire to kink and after which growth slows. Particles c and b both grow but at a slower overall rate. The effects of wetting, particle shape, particle size, and orientation may help explain why some nanowires do not grow beyound some limited range, and why there may be differences in average growth rates between different nanowire-particle couples.

	Ni	Ru	Cu	Pd	Pt	Ag	Au
R/r	1.56	1.40	1.60	1.22	1.48	1.87	0.86

Table 5.1 Average ratios of the diameter of different catalyst clusters over the diameter of corresponding Fe_3O_4 nanowires grown with these catalysts. The difference among different catalysts are caused by different wetting of the catalysts on the nanowires.
